# Study on the Performance and Efficiency of MgO/CuFeO_2_ Composite Catalyst in Degradation of Organic Pollutants

**DOI:** 10.3390/ma19194248

**Published:** 2026-10-08

**Authors:** Xuanhao Li, Junhua You, Jie Zhang

**Affiliations:** School of Materials Science and Engineering, Shenyang University of Technology, Shenyang 110870, China

**Keywords:** heterogeneous fenton-like catalysis, MgO/CuFeO_2_ composite, ofloxacin, heterojunction, reactive oxygen species

## Abstract

With the acceleration of industrialization and increasing human activities, antibiotic pollutants enter aquatic environments through various pathways and pose serious threats to ecological safety and human health. Ofloxacin (OFX), a typical fluoroquinolone antibiotic, is persistent in water media owing to its high chemical stability and poor biodegradability. In this study, MgO/CuFeO_2_-based Fenton-type composite catalysts were prepared to investigate their efficiency in removing OFX from water. Among the as-prepared materials, MgO/CuFeO_2_-20 wt% exhibited the optimum catalytic activity: under the conditions of 20 mg L^−1^ initial OFX concentration, 0.5 g L^−1^ catalyst dosage and 20 mM H_2_O_2_ at natural pH, it achieved approximately 95% ofloxacin removal within 120 min, with a pseudo-first-order kinetic rate constant k_1_ of 0.0461 min^−1^. Benefiting from MgO modification, the catalyst maintained high degradation efficiency over a broad pH range of 5–9 and retained satisfactory catalytic performance after five consecutive cyclic runs with only minor activity decay. Characterizations including XRD, SEM-TEM, XPS, BET, LSV-EIS and ESR-DMPO verified that the construction of tight MgO-CuFeO_2_ heterointerfaces accelerated interfacial electron transfer and promoted the regeneration of Cu^+^/Cu^2+^ and Fe^2+^/Fe^3+^ redox couples. Hydroxyl radicals (·OH) were identified as the dominant reactive oxygen species for pollutant oxidation. This work provides a facile modification strategy to alleviate particle aggregation and metal-ion leaching drawbacks of bare CuFeO_2_, offering experimental support for designing high-efficiency delafossite-based heterogeneous Fenton-like catalysts for antibiotic-containing wastewater remediation.

## 1. Introduction

Among various water treatment technologies, advanced oxidation technologies (AOTs) are considered one of the most promising deep treatment techniques due to their ability to generate reactive species with strong oxidation capabilities (such as ·OH, O_2_^·-^, 1O_2_, etc), which can mineralize organic pollutants into CO_2_, H_2_O, and harmless inorganic ions. Among them, photocatalysis and similar Fenton-like advanced oxidation technologies based on catalysts have attracted much attention due to their simple operation and high degradation efficiency [1,2,3,4,5,6,7]. However, a single catalyst often has limitations such as a narrow light response range, low charge separation efficiency, easy loss of active components, and limited pH applicability, which restricts its practical application performance. Therefore, designing and constructing a composite catalyst system with synergistic effects to enhance catalytic activity, stability, and applicability has become a current research hotspot in catalytic materials [8,9,10].

Magnesium oxide (MgO), as a typical metal oxide, has characteristics such as a large specific surface area, abundant surface hydroxyl groups, and many alkaline sites. It cannot only enrich pollutants in water bodies through adsorption but also serve as a catalyst carrier or a synergistic component to promote the generation and transfer of reactive species. In recent years, delafossite-type or delafossite-like composite metal oxides have shown excellent application potential in the field of catalysis due to their unique crystal structure, good redox properties, and chemical stability [11]. Among them, CuFeO_2_, as a typical p-type semiconductor material, has a suitable energy band structure and good photocatalytic activity. The presence of both Cu and Fe transition metal elements enables it to also play a synergistic catalytic role in the Fenton-like reaction, promoting the decomposition of oxidants such as H_2_O_2_ to generate highly active free radicals. Composing MgO with CuFeO_2_ is expected to effectively enhance the degradation efficiency and cycle stability of the catalyst for oxyfurazone through the synergistic effect between the two (such as interface charge transfer, adsorption-catalysis coupling, complementary active sites) and broaden its application prospects in actual water treatment [12,13,14,15].

Delafossite-type CuFeO_2_, a p-type bimetallic oxide semiconductor, combines favourable semiconductor band structure and abundant redox sites originating from co-existing Cu and Fe transition-metal centres [16,17,18]. In heterogeneous Fenton-like systems, the reversible Cu^+^/Cu^2+^ and Fe^2+^/Fe^3+^ redox couples can activate H_2_O_2_ to produce oxidative free radicals, making CuFeO_2_ a competitive candidate for degrading fluoroquinolone antibiotics [19,20,21]. Even so, bare CuFeO_2_ frequently undergoes serious nanoplate stacking, metal-ion leaching under acidic conditions, and restrained electron transfer, leading to decreased catalytic activity and reusability over repeated runs [22,23,24,25].

In this context, this work synthesizes MgO/CuFeO_2_ composite catalysts with gradient MgO loadings (15 wt%, 20 wt%, 25 wt%) by a sol–gel–calcination approach. Multiple characterizations, including XRD, SEM-TEM, XPS, BET and electrochemical measurements, are employed to analyze crystal phase, morphology, pore texture and interfacial electron-transfer behaviours. Batch degradation experiments targeting ofloxacin are carried out to evaluate catalytic performance, together with exploration of key operational parameters (H_2_O_2_ dosage, initial pH). Radical identification via ESR, control experiments and pseudo-first-order kinetic analysis are implemented to unravel the dominant catalytic mechanism and synergistic interactions between MgO and CuFeO_2_. Catalyst recyclability is further assessed through consecutive cyclic tests. This study aims to establish clear structure–performance relationships for MgO/CuFeO_2_ and deliver experimental basis and theoretical guidance for designing high-efficiency delafossite-based heterogeneous Fenton-like catalysts for antibiotic wastewater remediation.

## 2. Preparation of Catalyst and Experimental Plan

### 2.1. Preparation Method and Steps

Copper nitrate trihydrate (Cu(NO_3_)_2_·3H_2_O, ≥99.0%), iron nitrate nonahydrate (Fe(NO_3_)_3_·9H_2_O, ≥99.0%), magnesium nitrate hexahydrate (Mg(NO_3_)_2_·6H_2_O, ≥99.0%), citric acid monohydrate (chelating agent, ≥99.5%), hydrogen peroxide (H_2_O_2_, 30 wt%), ofloxacin (OFX, ≥98%), DMPO (5,5-dimethyl-1-pyrroline-N-oxide, spin-trapping agent, ≥97%) were all purchased from Sinopharm Chemical Reagent Co., Ltd. All chemicals were used as received without further purification. Deionized water was employed throughout all experimental procedures.

A MgO/CuFeO_2_ composite catalyst was prepared using the sol–gel method combined with calcination. First, a certain amount of copper nitrate (Cu(NO_3_)_2_·3H_2_O), iron nitrate (Fe(NO_3_)_3_·9H_2_O), and magnesium nitrate (Mg(NO_3_)_2_·6H_2_O) were dissolved in deionized water and stirred until a uniform solution was formed. Then, citric acid was added as a chelating agent, and the pH of the solution was adjusted to 7. The mixture was stirred and evaporated at 60 °C in a water bath until a gel was formed. The gel was dried in an oven at 105 °C for 12 h to obtain a dry gel precursor. The precursor was then placed in a muffle furnace and heated at a rate of 5 °C/min to temperatures 500 °C, where it was calcined for 4 h. After natural cooling, the material was ground and sieved to obtain MgO/CuFeO_2_ composite catalysts with different MgO loading levels (15 wt %, 20 wt %, 25 wt %). The MgO/CuFeO_2_ composites were fabricated by the sol–gel method followed by high-temperature calcination. The theoretical mass fraction of MgO (15 wt %, 20 wt %, 25 wt %) was precisely adjusted by tuning the molar dosage of magnesium nitrate precursor, while keeping the molar ratio of Cu:Fe fixed at 1:1 for the CuFeO_2_ phase.

#### Characterizing Instrument Model

X-ray Diffraction (XRD): Crystal-phase identification was performed on Rigaku SmartLab SE (Rigaku Corporation, Akishima, Tokyo, Japan).

Scanning Electron Microscopy (SEM): The morphology of the sample was observed using the Zeiss GeminiSEM 300 scanning electron microscope (Carl Zeiss AG, Oberkochen, Germany).

Transmission Electron Microscopy (TEM) & High-Resolution TEM (HRTEM) + elemental mapping: The TEM/HRTEM tests were conducted using the Thermo Scientific Talos F200X transmission electron microscope from the United States (Thermo Fisher Scientific, Waltham, MA, USA).

X-ray Photoelectron Spectroscopy (XPS): The XPS spectra were collected using the Japanese Shimadzu/Krayos AXIS Ultra DLD spectrometer (Shimadzu-Kratos Analytical, Manchester, UK).

N_2_ adsorption-desorption porosimetry (BET-BJH): Using the American Micromeritics ASAP 2460 analyzer (Micromeritics Instrument Corporation, Norcross, GA, USA), nitrogen adsorption tests were conducted under 77 K conditions to determine the specific surface area and pore size distribution.

Electron Spin Resonance (ESR): The activity oxygen species were detected at room temperature using the Brook A300 electron spin resonance spectrometer (Bruker BioSpin GmbH, Rheinstetten, Germany), with DMPO serving as the spin-trapping agent.

Electrochemical measurements (LSV and EIS): The electrochemical tests were conducted on the CHI-760E electrochemical workstation (Chenhua Instrument, Shanghai, China) using a standard three-electrode system. The ITO conductive glass loaded with the catalyst served as the working electrode, the platinum foil as the counter electrode, and the saturated calomel electrode (SCE) as the reference electrode; 0.1 mol/L sodium sulfate aqueous solution was used as the supporting electrolyte.

### 2.2. Experimental Plan

#### 2.2.1. Experiment on the Degradation of Pollutants by Catalysts

Using ofloxacin (OFX) as the target organic pollutant, catalytic degradation experiments were conducted in Fenton-like reaction system. The typical experimental procedure is as follows: a certain amount of MgO/CuFeO_2_ composite catalyst was added to 50 mL of an organic pollutant solution with a concentration of 20 mg/L. The mixture was first stirred in the dark for 30 min to achieve adsorption–desorption equilibrium. Then, a specific amount of oxidant (H_2_O_2_) was added to initiate the catalytic reaction. During the reaction, samples were collected at regular intervals, filtered through a 0.22 μm membrane. The pollutant concentration was calculated based on a standard calibration curve. The degradation efficiency (η) of the catalyst was calculated using the formula: η = (C_0_ − C)/C_0_ × 100%, where C_0_ is the initial concentration and C is the concentration at time t. The effects of factors such as initial pH (3–11) and H_2_O_2_ concentration on degradation efficiency were also investigated, and recycling experiments were performed to evaluate the catalyst’s stability and reusability.

#### 2.2.2. Catalyst Circulation Performance Test

To investigate the stability of the catalyst, the reacted catalyst from each experimental run was recovered, washed multiple times with distilled water and ethanol, and subsequently dried for subsequent recycling experiments under identical reaction conditions [26].

## 3. Results and Discussion

### 3.1. Structure and Morphology

**Figure 1:**
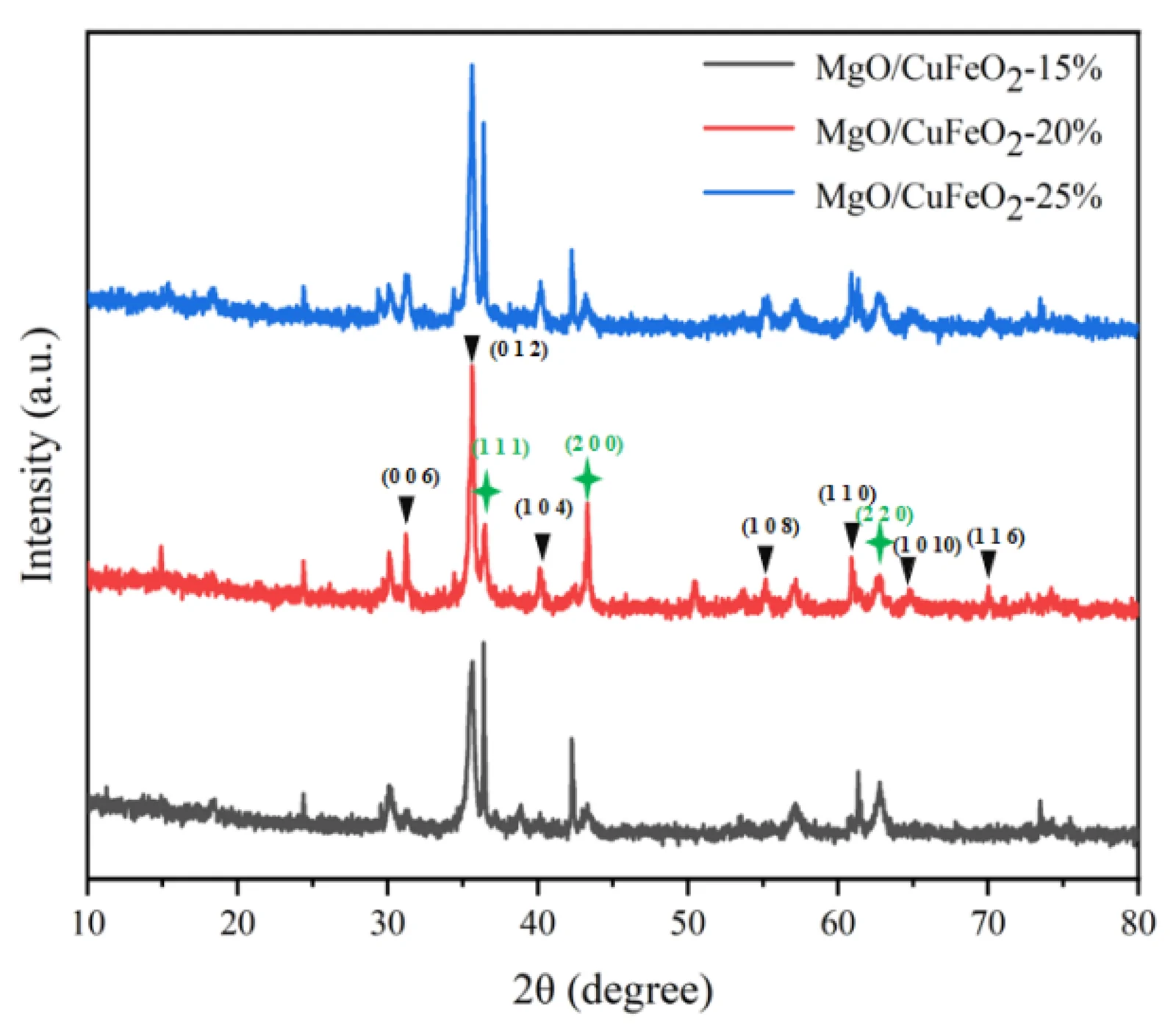
XRD patterns of MgO/CuFeO_2_ composite catalysts loaded with different proportions (The black inverted triangles denote the characteristic diffraction peaks of CuFeO_2_, and the green stars represent the MgO.).

Figure 1 presents the X-ray diffraction (XRD) patterns of MgO/CuFeO_2_ composite catalysts with three different mass loadings of MgO (15%, 20%, and 25%). The scanning 2θ range for the test is 10–80°. Inverted triangles indicate the characteristic diffraction peaks of CuFeO_2_, and green asterisks denote the characteristic diffraction peaks of MgO. All three groups of samples exhibit diffraction signals that fully match the standard card of CuFeO_2_ (JCPDS 39-0246), which correspond to all characteristic crystal planes of (006), (012), (104), (108), (110), (220), (1010), and (116). No impurity peaks assigned to CuO, iron oxides or other impurities are detected across the full diffraction spectrum, indicating that a high-purity CuFeO_2_ phase is successfully obtained via the preparation [27,28]. Meanwhile, the diffraction peaks corresponding to the (111), (200), and (220) crystal planes of MgO (JCPDS 45-0946) are detected in all samples, confirming the successful composite formation of MgO and CuFeO_2_.

The observed crystallinity variation is closely related to the incorporation mode and interfacial interaction between MgO and CuFeO_2_. At a low MgO loading of 15 wt %, dispersed MgO nanoparticles are anchored on the surface of CuFeO_2_ nanosheets to form heterogeneous interfaces. The limited amount of MgO imposes mild confinement on the crystal growth of CuFeO_2_, leading to relatively poor crystallinity. When MgO loading increases to 20 wt %, appropriate interfacial coupling between MgO and CuFeO_2_ optimizes the nucleation-and-crystallization behaviour during calcination, which facilitates the formation of well-crystallized composite heterostructures. Further increasing MgO content to 25 wt %, excess MgO tends to self-aggregate and undergoes obvious phase separation from CuFeO_2_. The abundant MgO-MgO grain boundaries reduce the overall system free energy and promote crystal growth of both phases, thus further improving the crystallinity of the composite. Nevertheless, excessive self-aggregation of MgO at high loading also covers part of the active heterointerfaces, which is detrimental to the catalytic performance. Such structural features well explain why MgO/CuFeO_2_-20 wt % delivers the optimal OFX-degradation activity rather than the highest crystallinity sample of 25 wt %.

#### 3.1.1. Catalyst Morphology

**Figure 2:**
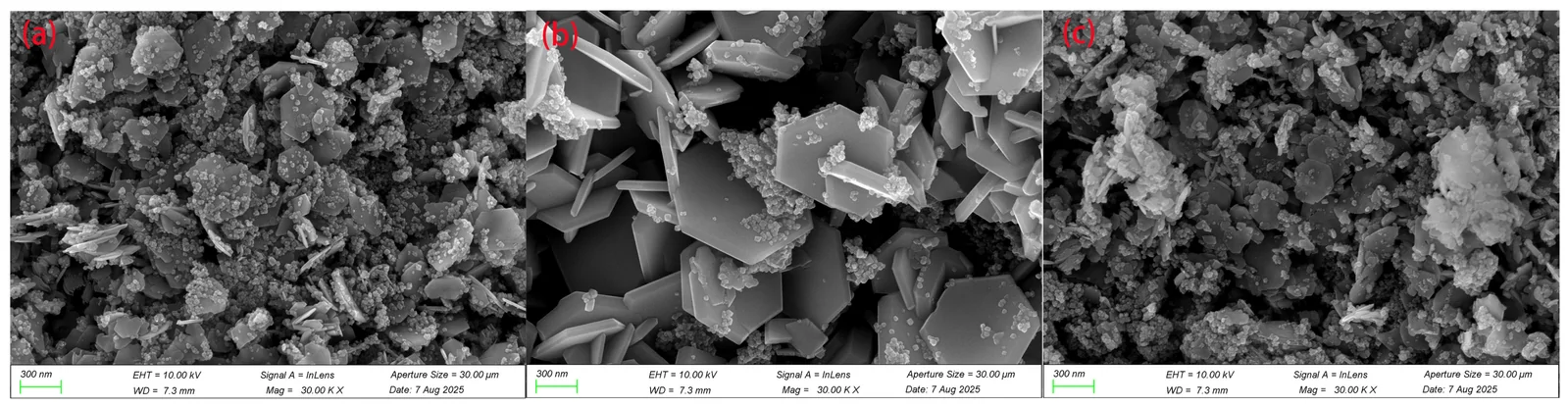
SEM images of MgO/CuFeO_2_ composite catalysts with different loading ratios: (**a**) 15%, (**b**) 20%, (**c**) 25%.

Figure 2a–c show the SEM morphologies of MgO/CuFeO_2_ composite catalysts with MgO loadings of 15%, 20% and 25%, respectively, acquired at a magnification of 30,000× (scale bar: 300 nm). The main bodies of the three groups of samples all exhibit the typical hexagonal sheet structure of delafossite CuFeO_2_, and a large number of MgO nanoparticles are uniformly attached to the surface of the hexagonal sheets [29,30,31].

For the MgO/CuFeO_2_-15% sample, the size of CuFeO_2_ sheets is relatively small, and the loading amount of MgO particles on the surface is low; for MgO/CuFeO_2_-20%, the hexagonal CuFeO_2_ sheets are well-crystallized with smooth surfaces, MgO nanoparticles are uniformly dispersed on the substrate surface without obvious agglomeration, and abundant two-phase contact interfaces are formed; when the MgO loading increases to 25%, a large number of MgO particles undergo agglomeration and accumulation, and the stacking of CuFeO_2_ sheets is aggravated [32]. The morphological changes indicate that the addition amount of MgO directly regulates the dispersion of the material: 20% MgO loading can achieve uniform dispersion of MgO and construct abundant heterogeneous interfaces; excessive loading (25%) tends to induce particle agglomeration and cover the catalytic active sites, which is not conducive to the contact between pollutants and oxidants. Abundant heterogeneous interfaces can accelerate electron transfer, which facilitates the generation of active free radicals in Fenton-like systems.

**Figure 3:**
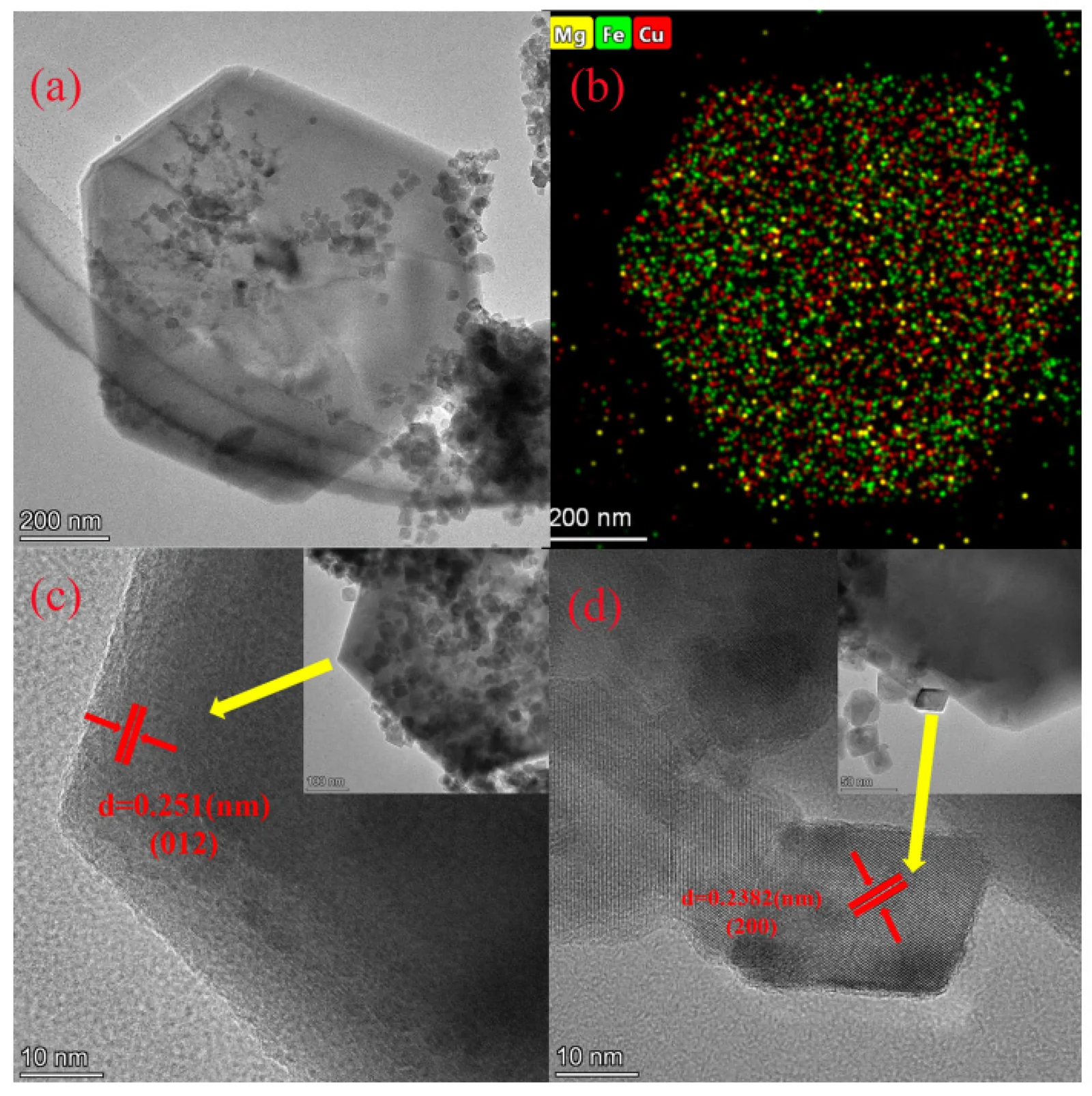
TEM image of the MgO/CuFeO_2_-20% Catalyst. (**a**) High-resolution transmission image of the MgO/CuFeO_2_ catalyst, (**b**) Mapping of MgO/CuFeO_2_ Catalyst, (**c**) The lattice stripes of CuFeO_2_, (**d**) The lattice stripes of MgO.

The MgO/CuFeO_2_-20% sample with the optimal overall morphology was selected for TEM, HRTEM and elemental mapping characterizations, and the results are presented in Figure 3. Hexagonal CuFeO_2_ nanosheets with a large number of MgO nanoparticles loaded tightly on their surface can be clearly observed in the low-magnification TEM image (Figure 3a). The elemental mapping results (Figure 3b) show that Mg, Fe and Cu are uniformly distributed and overlap across the entire hexagonal particle region, which verifies that MgO does not undergo local agglomeration and is uniformly loaded over the entire CuFeO_2_ substrate.

Two sets of clear lattice fringes can be observed in the high-resolution HRTEM images (Figure 3c,d): the lattice spacing of 0.251 nm corresponds to the (012) crystal plane of CuFeO_2_, while the lattice spacing of 0.2382 nm matches the (200) crystal plane of MgO [33,34]. The two sets of lattice fringes are closely adjacent, which directly demonstrates that MgO and CuFeO_2_ form a tightly bonded heterostructure. No lattice distortion is observed at the interface between the two phases, indicating that MgO is only loaded on the surface of CuFeO_2_ and does not enter the CuFeO_2_ lattice for doping [35], which is consistent with the XRD analysis results. The tight heterojunction interface shortens the electron transport path, accelerates the Cu^+^/Cu^2+^ and Fe^2+^/Fe^3+^ redox cycles, and thus improves the activation efficiency of H_2_O_2_.

#### 3.1.2. Xps Analysis of the Catalyst

**Figure 4:**
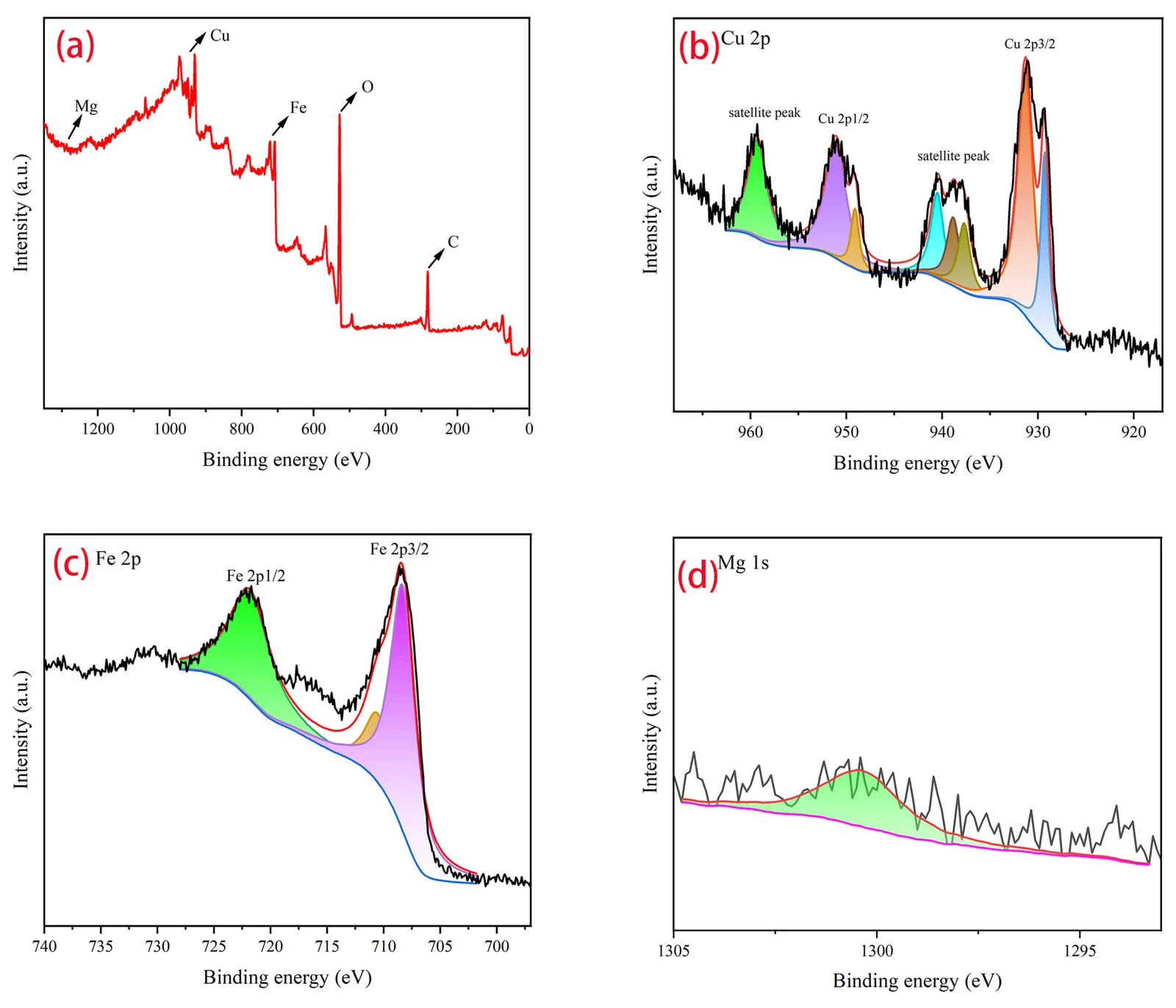
XPS survey spectrum of MgO/CuFeO_2_-20%. (**a**) The full spectrum of the MgO/CuFeO_2_ catalyst, (**b**) The Cu2p spectrum of the MgO/CuFeO_2_ catalyst, (**c**) The Fe 2p spectrum of the MgO/CuFeO_2_ catalyst, (**d**) The Mg 1s spectrum of the MgO/CuFeO_2_ catalyst.

Figure 4a presents the full XPS spectrum of the MgO/CuFeO_2_-20% catalyst acquired in the binding energy range of 0–1300 eV. The characteristic photoelectron peaks corresponding to Mg, Cu, Fe and O elements are clearly observed in the spectrum. The C peak originates from adventitious carbon introduced during measurement and is used for binding energy calibration. The co-detection of all target elements confirms that the catalyst contains both MgO and CuFeO_2_ components, which is consistent with the characterization results obtained from XRD and electron microscopy, further verifying the successful preparation of the composite catalyst [36,37]. Figure 4b–d show the high-resolution spectra of Cu, Fe and Mg, respectively. Peak fitting deconvolution of the Cu 2p high-resolution spectrum resolves the signal into Cu 2p_3_/_2_, Cu 2p_1_/_2_ and characteristic satellite peaks. Cu 2p_3_/_2_ is deconvoluted into peaks at 932.4 eV (corresponding to Cu^+^) and 934.8 eV (corresponding to Cu^2+^); Cu 2p_1_/_2_ corresponds to peaks at 952.2 eV (Cu^+^) and 954.5 eV (Cu^2+^). Two distinct satellite peaks located at 940–945 eV and 960–965 eV are characteristic signals of Cu^2+^, confirming that both monovalent copper and divalent copper coexist on the catalyst surface. The Cu^+^/Cu^2+^ redox couple serves as the core active site for Fenton-like activation of H_2_O_2_. Cu^+^ reduces H_2_O_2_ to generate hydroxyl radicals and is simultaneously oxidized to Cu^2+^; Cu^2+^ can be reduced back to Cu^+^ in the reaction system, enabling a cyclic redox process. The coexistence of the two valence states ensures the continuous cycling of copper active sites and maintains stable catalytic activity. For the Cu 2p spectrum, the Cu 2p_3_/_2_ peaks for Cu^+^ and Cu^2+^ are located at 932.4 eV and 934.8 eV, respectively, while the corresponding Cu 2p_1_/_2_ peaks appear at 952.2 eV (Cu^+^) and 954.5 eV (Cu^2+^). The spin–orbit splitting energy between Cu 2p_3_/_2_ and Cu 2p_1_/_2_ is approximately 19.8 eV, which is consistent with the reported value for copper-oxide-based materials. The characteristic satellite shake-up peaks of Cu^2+^ are observed at 940–945 eV and 960–965 eV, further verifying the coexistence of Cu^+^ and Cu^2+^ on the catalyst surface.

The Fe 2p spectrum consists of two major envelope peaks: Fe 2p_3_/_2_ (705–715 eV) and Fe 2p_1_/_2_ (718–728 eV). Peak deconvolution analysis reveals that surface iron exists as a mixture of Fe^2+^ and Fe^3+^: the peak at 710.1 eV in the Fe 2p_3_/_2_ region corresponds to Fe^2+^, while the peak at 712.4 eV is attributed to Fe^3+^; similarly, the peaks at 723.5 eV and 725.8 eV in the Fe 2p_1_/_2_ region correspond to Fe^2+^ and Fe^3+^, respectively [38]. The Fe^2+^/Fe^3+^ couple forms another redox cycle, which can synergistically interact with the copper cycle: Cu/Fe bimetallic systems mutually promote the regeneration of lower oxidation states, reduce the activation energy for H_2_O_2_ decomposition, enhance radical generation efficiency, and thereby achieve cooperative catalysis by the bimetallic system. In the Fe 2p high-resolution spectrum, Fe^2+^ and Fe^3+^ components of Fe 2p_3_/_2_ are centred at 710.1 eV and 712.4 eV, whereas the Fe 2p_1_/_2_ contributions for Fe^2+^ and Fe^3+^ occur at 723.5 eV and 725.8 eV. The spin–orbit splitting between Fe 2p_3_/_2_ and Fe 2p_1_/_2_ reaches around 13.4 eV, matching typical Fe-oxide reference data. In the Fe 2p high-resolution spectrum, Fe^2+^ and Fe^3+^ components of Fe 2p_3_/_2_ are centred at 710.1 eV and 712.4 eV, whereas the Fe 2p_1_/_2_ contributions for Fe^2+^ and Fe^3+^ occur at 723.5 eV and 725.8 eV. The spin–orbit splitting between Fe 2p_3_/_2_ and Fe 2p_1_/_2_ reaches around 13.4 eV, matching typical Fe-oxide reference data.

The main peak of the Mg 1s fine structure fitting is located near 1300 eV, matching the standard binding energy of Mg^2+^ in MgO, confirming successful loading of MgO onto the material surface. MgO provides basic sites for the catalyst, enriches H_2_O_2_ in aqueous solution, and modulates the electron cloud distribution on the CuFeO_2_ surface [39], thereby optimizing electron transfer efficiency at the heterojunction interface. Additionally, hydroxyl groups on the MgO surface can stabilize Cu^+^ and Fe^2+^ ions, reduce metal ion leaching, and enhance the catalyst’s cycling stability.

#### 3.1.3. The SBET and BJH Pore Size Distribution of the Catalyst Were Analyzed

**Figure 5:**
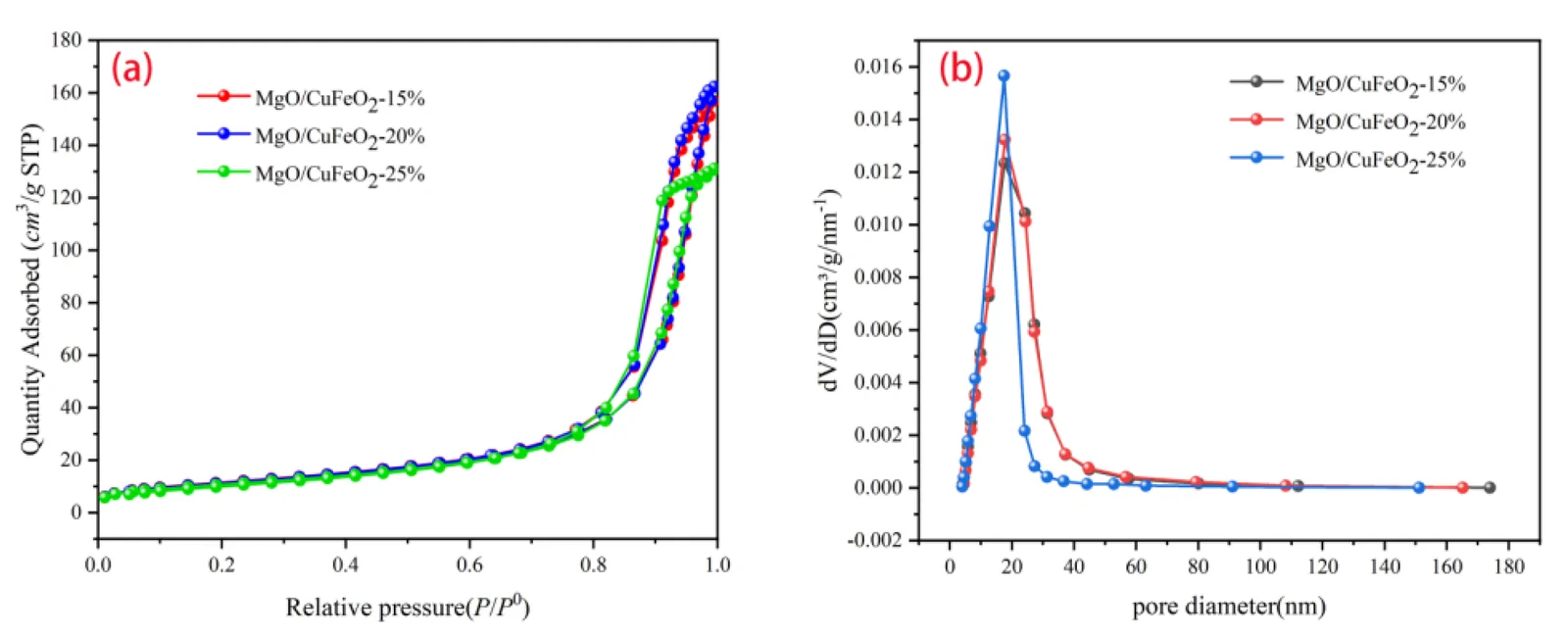
(**a**) N_2_ isothermal adsorption curves of MgO/CuFeO_2_ composite catalyst. (**b**) BJH adsorption average pore diameter of MgO/CuFeO_2_ composite catalyst.

The nitrogen adsorption–desorption isotherms of MgO/CuFeO_2_-15%, MgO/CuFeO_2_-20% and MgO/CuFeO_2_-25% catalysts are displayed in Figure 5a. All samples exhibit typical type-IV isotherms with obvious H3 hysteresis loops at P/P^0^ ranging from 0.8 to 1.0, confirming the existence of mesoporous structure derived from the stacking of aggregated nanoparticles [39]. The nitrogen uptake rises slowly at low relative pressure (P/P^0^ < 0.8), corresponding to the monolayer-multilayer adsorption of N_2_ on mesopore walls. A sharp increase in adsorption capacity is observed at high P/P^0^ (0.8–1.0), which originates from capillary condensation inside mesopores. The total adsorption volume follows the order MgO/CuFeO_2_-20% > MgO/CuFeO_2_-25% > MgO/CuFeO_2_-15%, demonstrating that 20% loading endows the catalyst with the largest specific surface area. Excessive CuFeO_2_ (25% loading) leads to partial pore blockage and structural collapse, reducing the adsorption capacity [40].

BJH pore size distribution curves (Figure 5b) reveal, the specific surface area and pore volume distribution are shown in Table 1, a represents the Brunauer-Emmett-Teller specific surface area, b represents the total pore volume, and c represents the average pore diameter. All catalysts possess narrow pore size distributions centred at 10–20 nm, confirming uniform mesoporous characteristics. The peak pore volume decreases in the sequence MgO/CuFeO_2_-20% > MgO/CuFeO_2_-15% > MgO/CuFeO_2_-25%. The maximum pore volume of MgO/CuFeO_2_-20% provides abundant mass transfer channels. For MgO/CuFeO_2_-25%, agglomeration of excess CuFeO_2_ blocks small mesopores, lowering pore volume and slightly increasing average pore diameter. In contrast, insufficient active sites limit the pore utilization of MgO/CuFeO_2_-15%.

The mesoporous texture is critical to the Fenton-like degradation of OFX. The high specific surface area and abundant mesopores of MgO/CuFeO_2_-20% expose massive Cu/Fe active sites, accelerating H_2_O_2_ activation to generate reactive oxygen species (·OH, O_2_^·-^) for OFX degradation. The interconnected mesoporous network reduces the diffusion resistance of OFX and H_2_O_2_, facilitating reactant access to active sites and desorption of degradation intermediates, thus mitigating catalyst surface fouling. The inferior pore structure of 15% and 25% samples restricts catalytic performance: low CuFeO_2_ loading supplies inadequate redox sites, while overloading causes particle aggregation and pore occlusion, embedding active species. Therefore, the optimized mesoporous structure of MgO/CuFeO_2_-20% accounts for its superior OFX degradation performance in the Fenton system.

### 3.2. Electrochemical Testing of Catalysts

**Figure 6:**
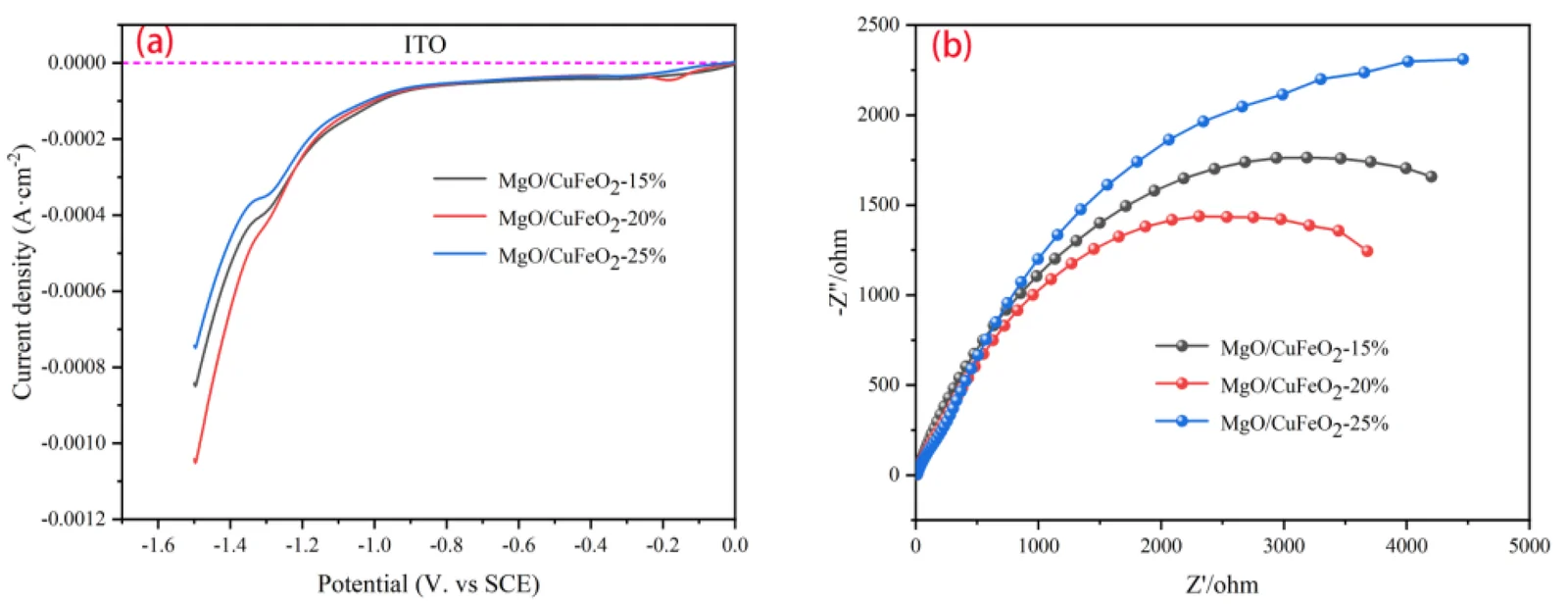
The LSV curve (**a**) and EIS curve (**b**) of the catalyst.

The electrochemical kinetics of three MgO/CuFeO_2_ catalysts were investigated via linear sweep voltammetry (LSV) and electrochemical impedance spectroscopy (EIS) experiments, and the corresponding results are presented in Figure 6. As observed from the LSV curves (Figure 6a), all catalysts exhibit distinct cathodic reduction currents, which originate from the electrochemical reduction of the Fe^3+^/Fe^2+^ and Cu^2+^/Cu redox couples on the catalyst surface [41]. These redox reactions dominate the cyclic transformation of metal species in heterogeneous Fenton reactions since Fe^2+^ and Cu are the key active sites for H_2_O_2_ activation.

Nyquist EIS plots (Figure 6b) show that the semicircle diameter corresponds to charge transfer resistance. A smaller semicircle means faster interfacial electron transfer and better electrochemical kinetics. The smallest arc of the 20% catalyst confirms the lowest charge transfer resistance. Uniform dispersion of MgO on CuFeO_2_ facilitates electrolyte penetration and tight electrode–electrolyte contact, accelerating electron migration. Aggregation in the 25% sample blocks mesopores, hinders electrolyte diffusion and increases electron transport resistance; the 15% sample suffers from poor conductivity owing to the low content of conductive Cu/Fe components.

### 3.3. The Catalytic Activity of the Catalyst

**Figure 7:**
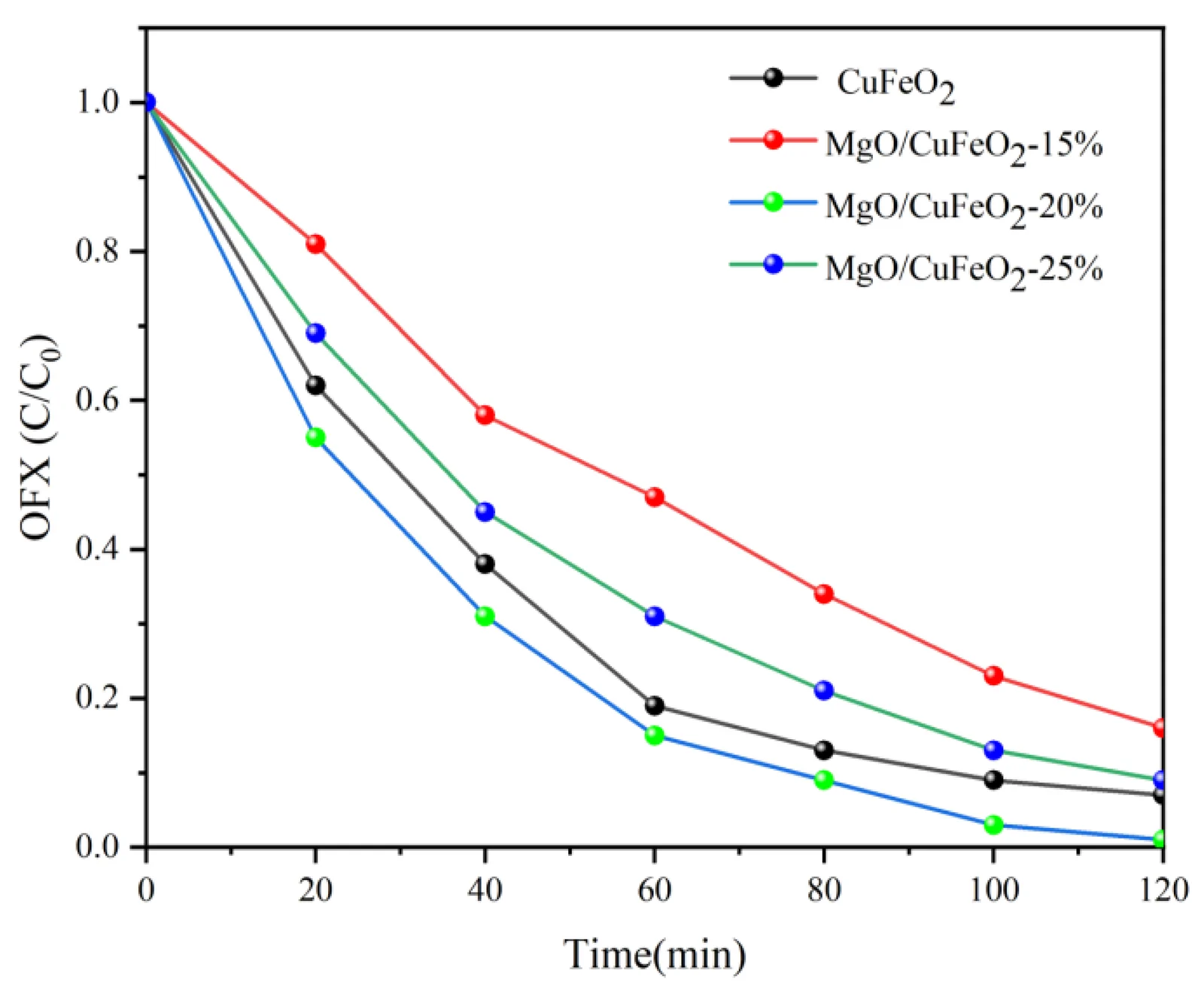
The degradation efficiency of OFX by the catalyst.

The degradation rates of OFX by catalysts of different proportions are shown in Figure 7, all curves continuously declined throughout the reaction. In the early stage, high substrate concentration and abundant free radicals led to rapid OFX degradation. In the later stage, as substrate decreased and intermediate products competed to quench free radicals, the degradation rate gradually slowed down, consistent with typical Fenton-like degradation kinetics. When the MgO loading is too high, the active component aggregates and blocks the pores, increasing charge transfer resistance and reducing the efficiency of metal redox cycles. The degradation performance is weaker than that of the 20% composite sample but still better than that of pure CuFeO_2_. When the MgO loading is insufficient, the total number of copper-iron sites capable of activating H_2_O_2_ is limited, resulting in restricted radical production.

**Figure 8:**
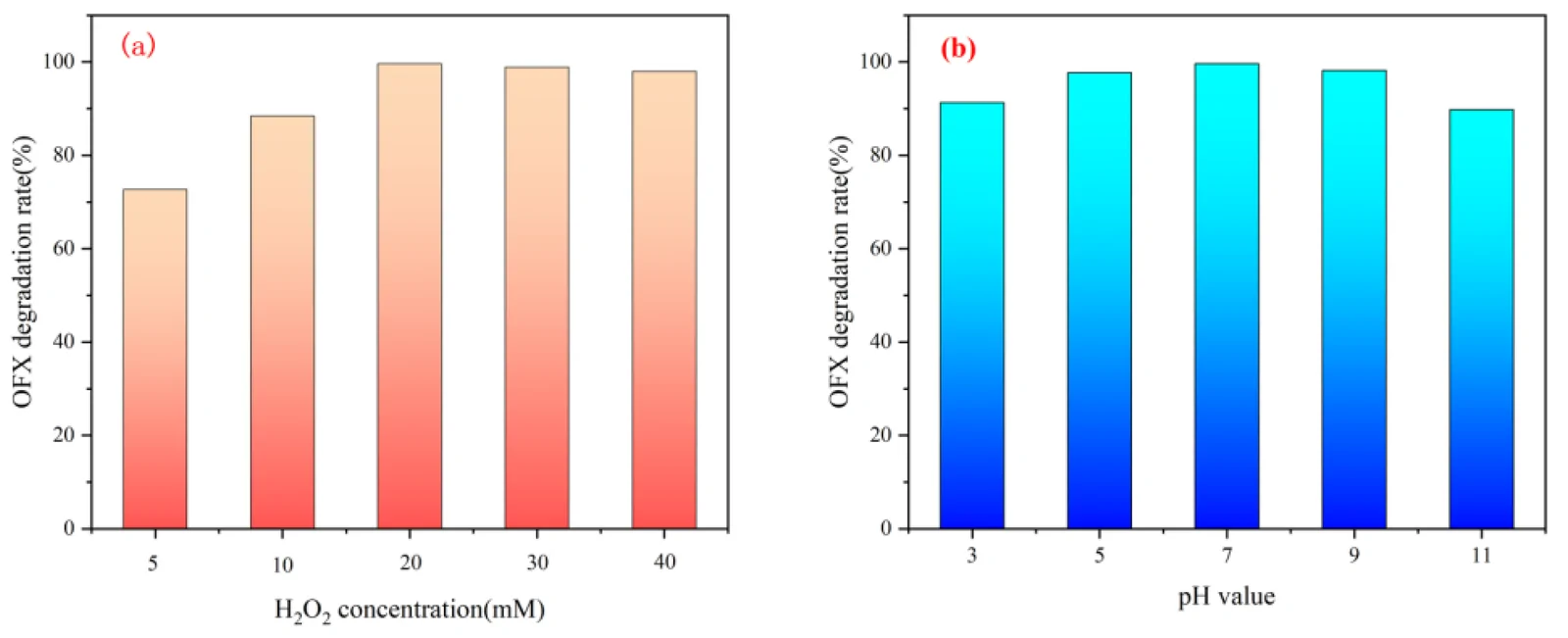
(**a**) Final residual proportion of OFX after 120 min reaction under different initial H_2_O_2_ concentrations. Conditions: MgO/CuFeO_2_-20%, initial OFX = 20 mg L^−1^, catalyst dosage = 0.5 g L^−1^, natural pH, room temperature. (**b**) Final residual proportion of OFX after 120 min reaction at different pH values. Conditions: MgO/CuFeO_2_-20%, initial OFX = 20 mg L^−1^, catalyst dosage = 0.5 g L^−1^, [H_2_O_2_] = 20 mM, room temperature.

The effects of H_2_O_2_ dosage and solution pH on OFX degradation were investigated using MgO/CuFeO_2_-20% (Figure 8). With H_2_O_2_ concentration increasing from 5 mM to 20 mM, the residual OFX ratio declined markedly owing to more ·OH generated from H_2_O_2_ activation; further raising H_2_O_2_ to 30–40 mM caused slight efficiency decrease because redundant H_2_O_2_ scavenges hydroxyl radicals. The catalyst exhibited the best degradation performance at neutral pH 7, while strong acid (pH = 3) led to metal leaching and poor activity, and strong alkali (pH = 11) induced rapid invalid decomposition of H_2_O_2_. Benefiting from MgO modification, the catalyst maintained high OFX degradation efficiency across pH 5–9, which greatly broadens the applicable pH window compared with conventional homogeneous Fenton.

### 3.4. The Cyclic Stability of the Catalyst

**Figure 9:**
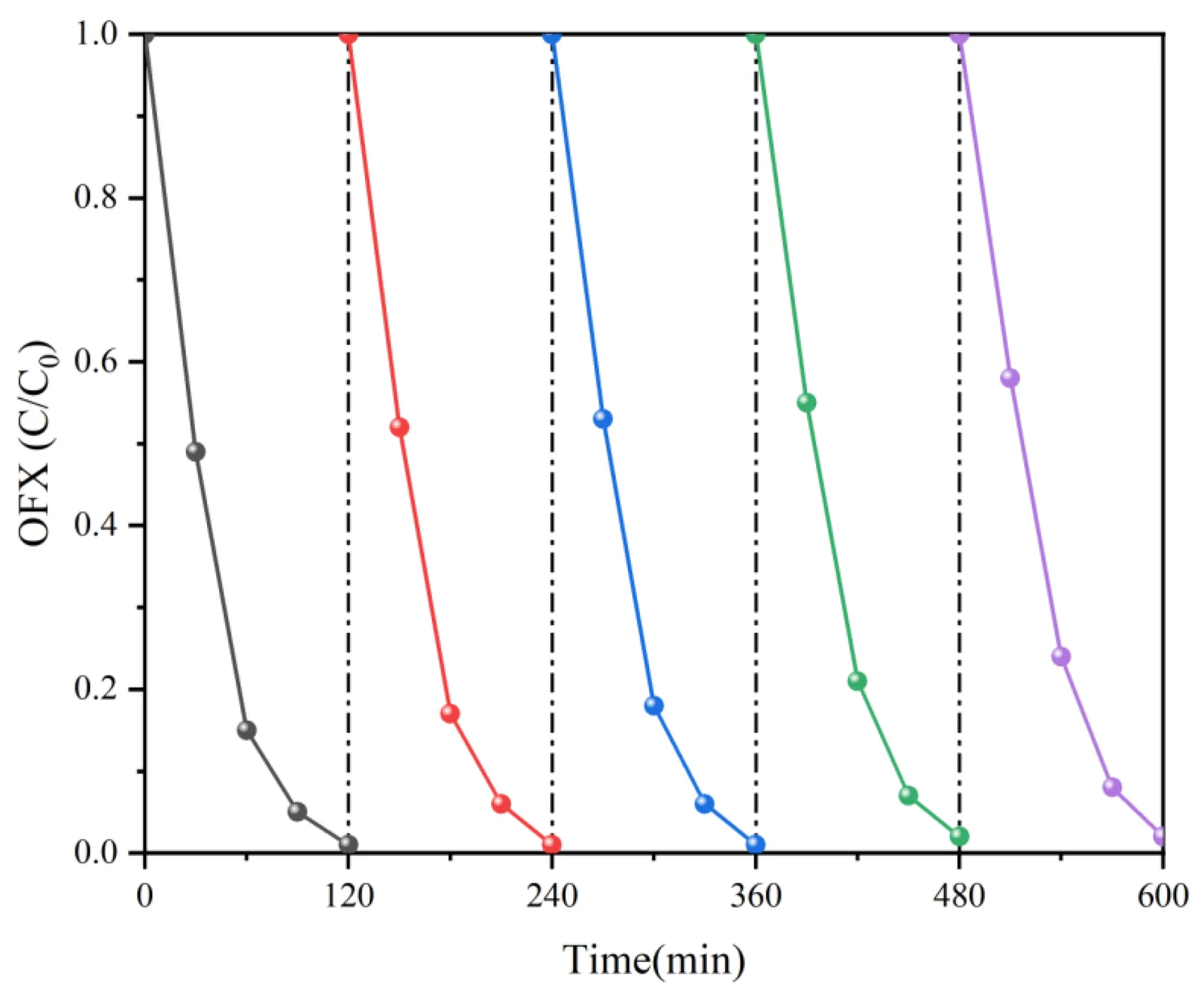
Degradation stability test of the catalyst (5 cycles, once every 120 min).

A catalyst should possess not only excellent activity but also superior stability. Accordingly, we conducted cyclic degradation tests on the MgO/CuFeO_2_-20% catalyst (Figure 9). During the cyclic degradation experiments, the MgO/CuFeO_2_-20% demonstrated outstanding stability, maintaining a high degradation efficiency for the target pollutant ofloxacin (OFX) with only marginal decline after multiple reuse cycles. These results indicate that the catalyst’s structure remains largely intact during repeated usage, with active sites effectively preserved. Combined with the aforementioned characterization data, it can be inferred that the unique morphology and chemical stability of the MgO/CuFeO_2_-20% constitute one of the key factors contributing to its sustained high catalytic performance.

### 3.5. The Degradation Efficiency of Different Catalysts

As shown in Table 2, bare CuFeO_2_ exhibits low kinetic rate constants and mainly works under acidic conditions, which limits its practical water-treatment application. Many modified CuFeO_2_ catalysts can improve degradation performance to a certain extent, but they still suffer from narrow pH tolerance or obvious metal-ion leaching during repeated usage. Benefiting from the constructed MgO heterostructure, MgO/CuFeO_2_-20% achieves a higher pseudo-first-order rate constant of 0.0461 min^−1^ under near-neutral conditions. It can maintain high degradation efficiency over a broad pH window of 5–9 and presents relatively stable recyclability. Although some reported catalysts can obtain satisfactory removal efficiency, our composite shows competitive comprehensive performance by balancing degradation kinetics, pH adaptability and anti-leaching ability.

### 3.6. Confirmation of Free Radicals in Catalytic Systems

**Figure 10:**
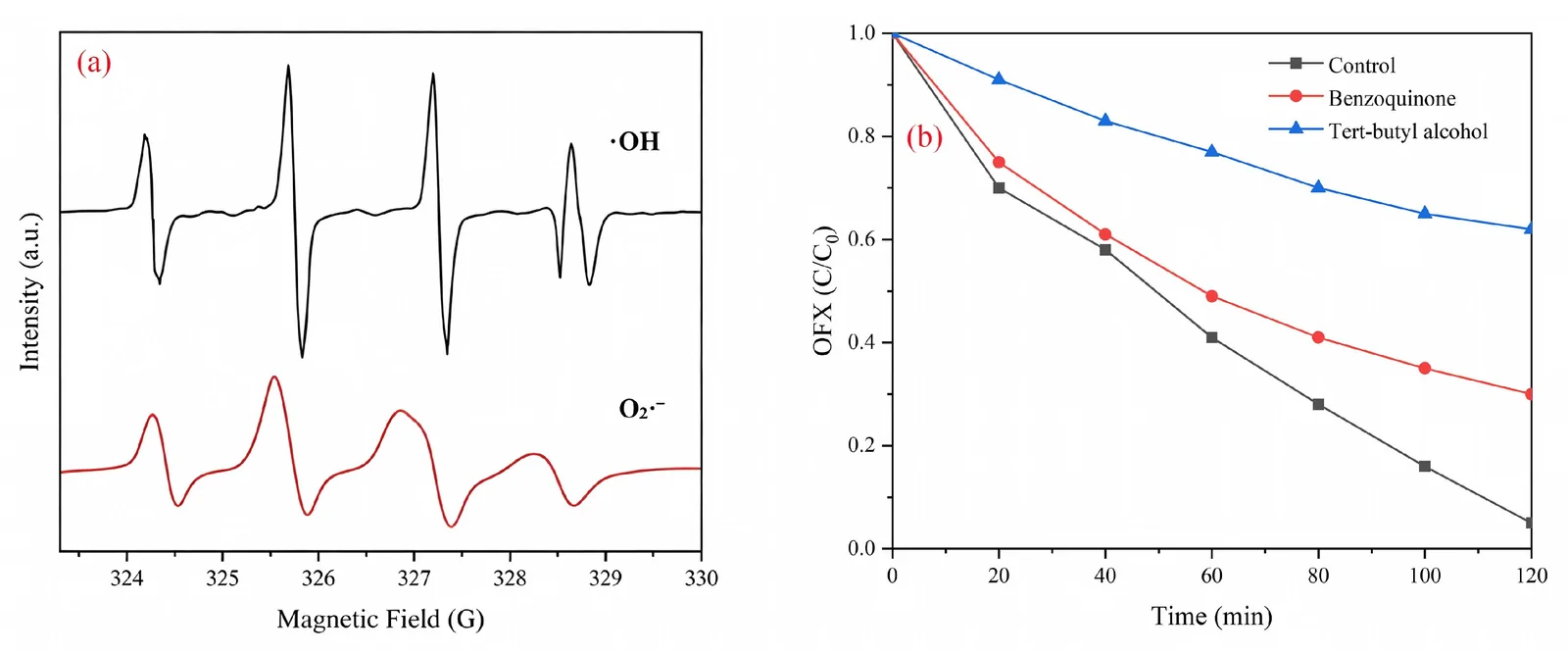
(**a**) ESR-DMPO spin-trapping spectra for •OH and O_2_^·−^; (**b**) OFX degradation profiles in the presence of different radical scavengers.

Figure 10a displays the ESR-DMPO spectra obtained for the MgO/CuFeO_2_-20% Fenton-like system. The typical four-line (1:2:2:1) characteristic signal of the DMPO-·OH adduct is clearly observed in the black curve, unambiguously confirming the generation of hydroxyl radicals within the catalytic system. Meanwhile, the red curve presents the fingerprint peaks assigned to DMPO-O_2_^·−^, verifying the simultaneous production of superoxide anion radicals. Evidently, the signal intensity of DMPO-·OH is much higher than that of DMPO-O_2_^·−^, implying that ·OH acts as the predominant reactive oxygen species, whereas O_2_^·−^ serves as a secondary radical participant during OFX degradation.

Radical quenching experiments were further carried out to quantify the contribution of each active species, as illustrated in Figure 10b. Without any scavenger (Control group), approximately 95% of OFX was eliminated within 120 min. After introducing tert-butyl alcohol (·OH scavenger), the degradation efficiency of OFX dropped markedly, and only ~38% OFX was removed after 120 min of reaction. Upon addition of benzoquinone (O_2_^·−^ scavenger), the degradation performance was moderately inhibited, and around 71% OFX was degraded at 120 min. The above quenching results are consistent with ESR observations, further demonstrating that ·OH dominates the oxidative degradation process, and O_2_^·−^ exerts an auxiliary role for OFX removal in the MgO/CuFeO_2_-20%-mediated Fenton-like reaction. Collectively, both ·OH and O_2_^·−^ participate in pollutant decomposition and jointly drive the degradation of ofloxacin.

### 3.7. Kinetic Analysis

Control-1: Catalyst only (no H_2_O_2_)

Reaction system: 50 mL ofloxacin (OFX) solution, catalyst dosage identical to formal runs, no addition of H_2_O_2_. Samples are collected at identical time intervals (0–120 min). This test quantifies pure adsorptive degradation of OFX onto catalyst surfaces under dark stirring.

Control-2: H_2_O_2_ only (no catalyst)

Reaction system: OFX solution + 20 mM H_2_O_2_, no solid catalyst. Evaluates direct OFX degradation by spontaneous H_2_O_2_ self-decomposition without catalyst activation.

Control-3: Heat-inactivated poisoned catalyst + H_2_O_2_

The catalyst is calcined at 400 °C for 6 h to deactivate surface redox sites. Run with H_2_O_2_ under standard conditions to rule out non-catalytic surface reactions.

Control experiment results show that direct oxidation by free H_2_O_2_ (Control-2) removes <6% OFX within 120 min, meaning spontaneous H_2_O_2_ decomposition contributes negligibly to pollutant elimination. Under catalyst-only conditions (Control-1), MgO/CuFeO_2_-20% achieves ~13% OFX degradation via surface adsorption after 120 min. After subtracting this adsorption fraction, the true catalytic degradation efficiency reaches ~87%, confirming that Fenton-like oxidation is the dominant pathway rather than physical adsorption.

Pseudo-first-order kinetic model ln(C_0_/C) = k_1_t was applied to quantify the OFX degradation kinetics (Table 3). The optimal MgO/CuFeO_2_-20% catalyst exhibited an apparent rate constant k_1_ = 0.0461min^−1^ with a high correlation coefficient R^2^ = 0.9981, verifying that OFX elimination well obeyed pseudo-first-order kinetics. The kinetic constants followed the order MgO/CuFeO_2_-20% > CuFeO_2_ > MgO/CuFeO_2_-25% > MgO/CuFeO_2_-15%, consistent with degradation curves. Residual analysis showed a low total residual sum of squares, and most experimental points fitted the straight line well; slight residual deviation at the early reaction stage originated from the explosive generation of reactive radicals, while later-stage data presented better linearity. The standard error of k_1_ was only 0.00072, demonstrating high reliability of kinetic parameters [42]. The relative standard deviation (RSD) of parallel tests was 2.93% (Table 4), indicating acceptable experimental repeatability. The errors mainly came from HPLC detection fluctuations, temperature disturbances, inevitable pH drift and model inherent limitations since the actual heterogeneous Fenton reaction involved complicated radical chain reactions and intermediate competitive consumption. With recycling cycles increasing, k_1_ gradually declined and RSD rose mildly due to trace metal leaching and surface intermediate adsorption, yet all cycles maintained R^2^ > 0.996, confirming persistent compliance with pseudo-first-order kinetics.

All catalytic degradation experiments were conducted in triplicate (*n* = 3). Rate constants k_1_ are reported as mean ± standard deviation (SD). Relative standard deviation (RSD < 4.1%) demonstrates acceptable experimental reproducibility. One-way ANOVA analysis shows that the k_1_ of MgO/CuFeO_2_-20% is statistically significantly higher (*p* < 0.05) than all other catalyst groups. The gradual decrease in k_1_ over five cycles is statistically significant (*p* < 0.05), attributed to trace metal leaching and surface adsorption of degradation intermediates.

### 3.8. Discussion on Reaction Mechanism

**Figure 11:**
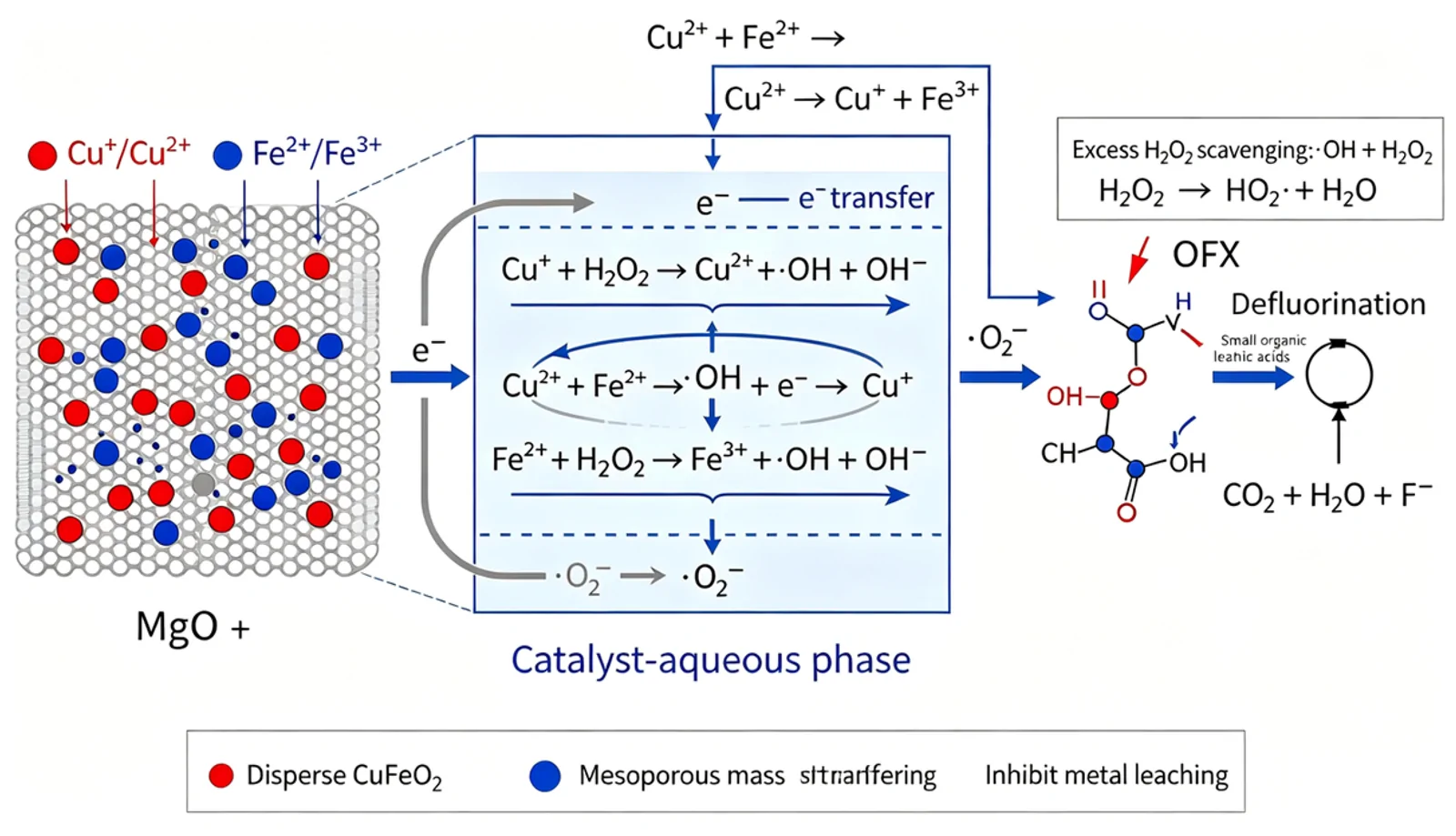
The reaction mechanism of a catalyst.

The schematic diagram illustrates the reaction pathway for OFX degradation via MgO-modified CuFeO_2_-based Fenton-like catalysis (Figure 11). CuFeO_2_ nanoparticles serve as the primary catalytic framework, inherently containing redox-active sites of Cu^+^/Cu^2+^ and Fe^2+^/Fe^3+^, while MgO is uniformly anchored on the surface of CuFeO_2_ particles. Bidirectional interfacial electron transfer occurs between Cu and Fe sites, with electron shuttling between copper and iron accelerating the regeneration cycle of lower-valent species Cu^+^ and Fe^2+^. The low-valent metal sites activate H_2_O_2_ to generate hydroxyl radicals (·OH) and superoxide radicals (·O_2_^−^), which attack the aromatic ring, C–F bonds, and piperazine side chains of OFX, leading to defluorination and ring-opening oxidation. OFX is progressively decomposed into small organic acids and ultimately mineralized into harmless products such as CO_2_, H_2_O, fluoride ions, and nitrate. The MgO attached to the CuFeO_2_ surface plays multiple regulatory roles: the outer MgO layer isolates adjacent CuFeO_2_ particles, preventing aggregation and ensuring full exposure of internal Cu and Fe active sites; the mesoporous MgO thin film enhances the diffusion of H_2_O_2_ and OFX from aqueous solution to the inner CuFeO_2_ active sites, while also promoting desorption of degradation intermediates to prevent site blockage and deactivation by pollutants; additionally, MgO forms a weakly alkaline interface that buffers the overall pH, reducing precipitation of copper and iron hydroxides and broadening the effective pH range for Fenton reactions. A side reaction shown in the figure involves excess H_2_O_2_ consuming ·OH to form less reactive HO_2_·. A 20% loading ratio of MgO is optimal—lower loadings provide insufficient protective dispersion, whereas higher loadings form dense barrier layers that hinder reactant access to internal active sites and extend electron transfer pathways, resulting in reduced catalytic activity.

## 4. Conclusions

In this work, MgO surface-decorated CuFeO_2_ catalysts with three MgO loadings (15%, 20%, 25%) were synthesized via sol–gel calcination, and systematic characterizations including XRD, SEM, TEM, XPS, BET, LSV and EIS verified that MgO nanoparticles uniformly attach to hexagonal CuFeO_2_ nanosheets to form compact heterointerfaces without lattice doping, among which MgO/CuFeO_2_-20% possesses the largest specific surface area and pore volume, optimal mesoporous structure, minimal charge transfer resistance and fastest interfacial electron migration between coexisting surface Cu^+^/Cu^2+^ and Fe^2+^/Fe^3+^ redox pairs; batch OFX degradation, ESR radical identification and pseudo-first-order kinetic tests confirmed the activity order MgO/CuFeO_2_-20% > bare CuFeO_2_ > MgO/CuFeO_2_-25% > MgO/CuFeO_2_-15%, the optimal 20% composite achieved nearly complete OFX degradation within 120 min at 20 mM H_2_O_2_ and neutral pH with a maximum rate constant k_1_ = 0.0461 min^−1^ and high R^2^ = 0.9981, surface MgO endows the catalyst with a broad applicable pH window of 5–9 and favourable recyclability over five cycles with only slight activity decay induced by trace metal leaching and intermediate adsorption, and the reaction mechanism clarifies that bidirectional Cu–Fe electron shuttle accelerates the regeneration of low-valence active metals to continuously activate H_2_O_2_ while surface MgO disperses CuFeO_2_, facilitates mass transfer, buffers interfacial pH and suppresses metal dissolution, excessive MgO coating blocks active sites and insufficient decoration fails to restrain particle aggregation, both deteriorating catalytic performance, and this MgO surface modification strategy effectively addresses the defects of bare CuFeO_2_, including severe agglomeration, narrow pH adaptability and poor reusability, provides a simple preparation method and theoretical guidance for the application of heterogeneous Fenton catalysts in the purification of actual antibiotic wastewater.

## Figures and Tables

**Table 1:** Parameters of different samples obtained from the analysis of N_2_ adsorption isotherms.

Samples	SBET (m^2^ g^−1^) ^a^	Pore Volume (cm^3^ g^−1^) ^b^	Average Pore Size (nm) ^c^
CuFeO_2_	19.8653	0.0934	30.6423
MgO/CuFeO_2_-15%	40.1104	0.2433	21.6035
MgO/CuFeO_2_-20%	43.651	0.2517	22.241
MgO/CuFeO_2_-25%	41.3757	0.2022	18.5312

**Table 2:** Comparison of degradation efficiency of other CuFeO_2_-based catalysts.

Catalyst	Oxidant	OFX Initial Concentration (mg L^−1^)	Catalyst Dosage (g L^−1^)	pH Working Range	OFX Removal Efficiency	Ref.
MgO/CuFeO_2_-20% (This work)	H_2_O_2_	20	0.5	5–9	95%	This work
Bare CuFeO_2_	H_2_O_2_	10	1.1	Only acidic favourable; performance decreases greatly at pH > 6	93.8%	[2]
CuFeO_2_@PVP	photo-electro-Fenton	10	0.4	Only effective under acidic pH	94.3%	[4]
Biochar/CuFeO_2_	H_2_O_2_	20	0.6	4–7	88.2%	[1]
g-C_3_N_4_/CuFeO_2_	H_2_O_2_	20	0.5	4–8	90.1%	[9]
Oxygen-vacancy CuFeO_2_	H_2_O_2_	15	0.5	5–8	92.5%	[12]

**Table 3:** Pseudo-first-order kinetic fitting results for different catalysts.

Catalyst Morphology	Pseudo-First-Order Rate Constant k_1_ (min^−1^)	Linearly Dependent Coefficient R^2^
CuFeO_2_	0.0274 ± 0.0008	0.9963
MgO/CuFeO_2_-15%	0.0201 ± 0.0006	0.9970
MgO/CuFeO_2_-20%	0.0461 ± 0.0014	0.9981
MgO/CuFeO_2_-25%	0.0247 ± 0.0007	0.9966

**Table 4:** k_1_ and R^2^ after five cycles.

Cycle Index	Pseudo-First-Order Rate Constant k1 (min^−1^)	Linearly Dependent Coefficient R^2^	RSD
1	0.0461 ± 0.0014	0.9981	2.93%
2	0.0452 ± 0.0013	0.9979	2.88%
3	0.0443 ± 0.0012	0.9977	2.93%
4	0.0434 ± 0.0012	0.9974	2.76%
5	0.0426 ± 0.0011	0.9972	2.82%

## Data Availability

The original contributions presented in this study are included in the article. Further inquiries can be directed to the corresponding author.
